# Racial Differences in Associations of Cognitive Health Status With Happiness, Helplessness, and Hopelessness Among Older Adults: An Exploratory Study

**DOI:** 10.3389/fnagi.2022.890404

**Published:** 2022-05-11

**Authors:** Emre Umucu, Beatrice Lee, Mary Wyman, Diane Carol Gooding, Carol Ann Van Hulle, Adrienne Johnson, Carola A. Ferrer Simo, Fabu Carter, Hector Salazar, Taryn T. James, Shenikqua Bouges, Nicholas H. Lambrou, Sterling C. Johnson, Sanjay Asthana, Carey E. Gleason

**Affiliations:** ^1^Department of Counseling, Educational Psychology and Special Education, Michigan State University, East Lansing, MI, United States; ^2^W. S. Middleton Memorial Veterans Hospital, Madison, WI, United States; ^3^School of Medicine and Public Health, University of Wisconsin-Madison, Madison, WI, United States; ^4^Alzheimer’s Disease Research Center, University of Wisconsin-Madison, Madison, WI, United States; ^5^Department of Psychology, University of Wisconsin-Madison, Madison, WI, United States; ^6^Center for Tobacco Research and Intervention, University of Wisconsin School of Medicine and Public Health, Madison, WI, United States; ^7^Division of Geriatrics, Department of Medicine, The School of Medicine and Public Health (SMPH), University of Wisconsin-Madison, Madison, WI, United States; ^8^Geriatric Research, Education and Clinical Center, William S. Middleton Memorial Veterans Hospital, Madison, WI, United States

**Keywords:** happiness, helplessness, hopelessness, MCI, dementia, race

## Abstract

**Background:**

The relationship between healthy and positive aging and dementia and cognitive impairment has received limited attention in the field of aging. Affect impacts cognitive changes and processes, and cognitive impairment is associated with affective comorbidities. The purpose of the study was to examine (a) whether happiness, helplessness, and hopelessness are linked to cognitive health status, and (b) whether these associations differ by race.

**Methods:**

Participants were enrollees in the Wisconsin Alzheimer’s Disease Research Center’s Clinical Core (ADRC). Average age at baseline was 60.85 (*SD* = 8.65), 73.70 (*SD* = 8.02), and 73.80 (*SD* = 9.59) years for cognitively normal individuals, individuals with MCI, and individuals with dementia, respectively.

**Results:**

In the full sample, chi-square test results revealed associations between Cognitive Health Status (CHS) and (a) happiness, χ^2^(2) = 6.06, *p* < 0.05, (b) helplessness, χ^2^(2) = 6.44, *p* < 0.05, and (c) hopelessness, χ^2^(2) = 14.11, *p* < 0.01.

**Conclusion:**

This study provides support for the association of both positive and negative affect with cognitive health status in middle- to older-aged adults.

## Introduction

Mild cognitive impairment (MCI) is an early stage of memory loss or other cognitive ability loss in people maintaining the ability to independently perform most of their daily life activities (Alzheimer’s Association, 2022). In their systematic review and data synthesis study, Gillis and colleagues reported “meta-analysis estimates (95% CI) of MCI incidence per 1,000 person-years were 22.5 (5.1–51.4) for ages 75–79y, 40.9 (7.7–97.5) for ages 80–84y, and 60.1 (6.7–159.0) for ages 85+y” (p. 248) (Gillis et al., 2019). Individuals with MCI may develop dementia (Alzheimer’s Association, 2022), defined as the loss of cognitive functioning (e.g., thinking, reasoning, and remembering) that interferes with individuals’ daily life and activities (National Institute on Aging, 2021). According to Alzheimer’s Association (Alzheimer’s Association, 2022), majority of MCI cases result from brain changes occurring in very early stages of Alzheimer’s disease or other neurodegenerative diseases that cause dementia.

Research on the relationship between healthy and positive aging (e.g., optimal, successful, productive, and healthy aging; Bar-Tur, 2021) and dementia and cognitive impairment has been relatively overlooked in the field of aging. Given the bidirectional relationship between cognitive and affective health, it is important to examine the interplay of these factors to better understand how emotional health and positivity in the context of aging influence the presentations of dementia and cognitive impairment. For example, affective perturbations impact cognitive processes, and cognitive impairment is associated with affective comorbidities (Butters et al., 2008; Dolcos et al., 2011; Sutin et al., 2018). Changes in positive and negative affect are linked to incident cognitive impairments in older adults (Zank and Leipold, 2001; Rickenbach et al., 2015; Sutin et al., 2018). Likewise, severity of dementia is associated with alterations in affect (Zank and Leipold, 2001). Given the inter-relatedness of affect and cognitive symptoms, and their mutual dependence on brain health, it is important to further explore these associations in older adults across a range of cognitive statuses, including cognitively healthy individuals, those with mild cognitive impairment (MCI), and those with dementia.

Positive affect refers to emotional experiences related to positive interactions with the environment and comprises elements such as enthusiasm and joy (Clark et al., 1989). Schreiner et al. (2005) found that persons with dementia report less positive affect than cognitively healthy individuals. Another study found that people with moderate Alzheimer’s disease were able to experience happiness (Shell, 2015), suggesting preservation of positive affect despite significant neurodegeneration. In contrast to positive affect, negative affect refers to subjective distress and comprises negative states such as anger and disgust (Clark et al., 1989). Negative affect includes helplessness and hopelessness (Shields, 1992).

In general, greater negative affect has been found to be associated with increased risk of cognitive impairment (Håkansson et al., 2015; Korthauer et al., 2018; Sutin et al., 2018). Gates et al. (2014) found an inverse association between cognitive function and negative affect in community-dwelling adults diagnosed with MCI. For example, their results revealed that the greater levels of memory complaints were associated with more depressive symptoms (Gates et al., 2014). Danhauer et al. (2013) found that negative affect was associated with reduced global cognitive function. Feelings of helplessness were found to be associated with more severe memory deficits in people with dementia (van Wanrooij et al., 2019). In another study, researchers found that greater awareness of cognitive impairment is associated with feelings of hopelessness (Harwood and Sultzer, 2002)—perhaps suggesting a mechanism for mood comorbidities associated with neurodegenerative disorders. In total, alterations in affect and cognition may overlap. However, more future research is warranted to test this thesis.

Previous studies have examined whether there are cultural differences in the expression of positive and negative affect, examining racial groups. In an exploratory study examining older adults with cancer, researchers found that Black participants endorsed greater positive affect (i.e., excitement, inspiration, determination, alertness, attentiveness) compared to White participants (Krok-Schoen and Baker, 2014). The researchers explained older Black adults having more internal and external resources (e.g., resiliency, social support) to cope with the adverse effect of cancer and aging than older White adults (Gates et al., 2014). The authors also reported that Black participants may have a more supportive kinship network than Whites (Krok-Schoen and Baker, 2014). It is also possible, that assessment of mood is biased, leading to under or over-reporting based on race or culture. Using data from the MIDUS (Midlife in the United States) sample, Lankarani and Assari (2017) found that Blacks reported higher levels of positive affect than Whites in the presence of comparable negative affect leader to a weaker negative association between positive affect and negative affect, net of demographic, socioeconomic, and health status (Lankarani and Assari, 2017).

Investigations examining differences in negative affect between Whites and Blacks yielded mixed findings. One study revealed no group differences in hopelessness in college students, though this may be attributable to the individuals having adaptive resources given that they were successfully enrolled in college (Hirsch et al., 2012). A more recent study found that the association between baseline chronic health conditions and changes in negative affect was stronger for White participants compared to Black participants (Assari and Lankarani, 2016). Authors reported that further research should explore depression and chronic medical conditions (e.g., diabetes, cancer) among Whites and Blacks (Assari and Lankarani, 2016). Authors also emphasized that further research is needed to identify types of chronic medical conditions that may differently associate with negative affect (Assari and Lankarani, 2016). In another research, authors discussed possible Black and White participants differences in reciprocal links between negative affect and chronic medical conditions over time, characterizing a Black-White health/mood paradox (i.e., “less frequent depression despite a higher prevalence of chronic medical conditions among Blacks compared to Whites in the United States; Assari et al., 2015).” This unexpected relationship between mood and health emerges in mid and later life (Assari et al., 2015). Similarly, our recent work found older White females were more likely to indicate role impairment due to depression than older Black females even when depression severity is similar, which the authors hypothesized that there could be due to racial differences in illness perception, health appraisals, and health behaviors (Wyman et al., 2019). The authors also noted that racial differences in the DSM-IV clinical significance criterion endorsement are not due to health factors or depression symptom presentation; However, it could be due to clinicians’ under-diagnosis of depression among Black older adults.

Overall, the literature suggests that brain changes associated with neurodegenerative conditions result in affective and cognitive alterations, and that affective expression and associations may vary by race. Relatively few studies have examined whether unique dimensions of positive (i.e., happiness) and negative affect (i.e., helplessness and hopelessness) are associated with cognition across a range of cognitive health status (CHS), and whether these relationships differ by race. The aim of this exploratory study was to address this question using a well-characterized sample consisting of cognitively healthy individuals, those with MCI, and those with dementia.

## Materials and Methods

### Procedure

Data were extracted from the Wisconsin Alzheimer’s Disease Research Center’s Clinical Core (ADRC) data. Wisconsin ADRC longitudinally follows participants; However, for the current cross-sectional study, only participants’ baseline data were used. Participants were recruited from community and memory clinics (Ma et al., 2021). After provided informed consent, participants completed clinical and cognitive assessments. The study and all procedures were approved by the Institutional Review Board at the University of Wisconsin-Madison.

This study included participants with no impairment, MCI, and dementia based on their baseline diagnosis. CHS was established through consensus conference determination based on comprehensive evaluation data. Consensus conferences are attended by neuropsychologists, geriatricians, neurologists, and team members collecting the data, including nurse practitioners and cognitive testers. Participants whose CHS could not be adjudicated as one of the three categorical states were excluded, e.g., those determined to have cognitive impairment but did not meeting clinical criteria for MCI or dementia. Total remaining sample included 755 participants. ADRC’ detailed clinical diagnosis procedure is described elsewhere (Blazel et al., 2020; Ma et al., 2021).

### Measures

We obtained participants’ demographic characteristics (i.e., age, sex, education, and race) from self-report questionnaires. Race is a sociocultural construct. In this study we examine differences based on the experience of being racialized as Black or as White, asking participants to describe their racial identity using categories used by the US Census. We also asked participants to indicate ethnicity non-Hispanic vs. Hispanic.

Mood states were derived from items included in the 15-item *Geriatric Depression Scale* (GDS) (Sheikh and Yesavage, 1986). In particular, happiness was assessed by asking participants to respond with a yes or no in reference to how they felt over the past week. The single item included the question: “Do you feel happy most of the time?” Hopelessness and helplessness were assessed with 2 items of the GDS (Sheikh and Yesavage, 1986): “Do you often feel helpless?” and “Do you feel that your situation is hopeless?.” Numerous studies have used single items to measure these constructs (Yu and Chen, 2016; Fortuna et al., 2020; Lu et al., 2021).

### Data Analysis

Descriptive statistics were calculated to provide information regarding participant’s demographic and clinical characteristics. For our first purpose (i.e., whether happiness, helplessness, and hopelessness are linked to cognitive health status), participants from all racial groups were combined in the analyses. To explore our second study goal (i.e., whether these associations differ by race), we included only participants who self-identified their primary race as either Black or White (*N* = 732) and excluded participants from other racial groups. Chi-square or Fisher-Freeman-Halton Exact tests were conducted to test our research hypotheses. We used Cramer’s V to provide a measure of effect size. All analyses were conducted using SPSS 28.0.

## Results

### Descriptive Statistics

Descriptive statistics are reported in Table 1. The mean age at baseline was 60.85 years (*SD* = 8.65) for cognitively normal individuals. The mean age at baseline was 73.70 years (*SD* = 8.02) for MCI group. The mean age at baseline was 73.80 years (*SD* = 9.59) for dementia group. Regarding participants’ gender, 66.4% of cognitively normal individuals, 34.8% of MCI group, and 42.2% of dementia group were female. Majority of our participants in each group were White (79% for cognitively normal group, 87.6% for MCI group, and 89% for dementia group). Our results also revealed that there were significant relationships between CHS and gender, independence status, living status, and race. Women, those who live independently, and those living alone were more likely to be cognitively healthy than their counterparts.

**TABLE 1:** Baseline characteristics of participants.

Characteristics	Cognitively normal *n* = 557	Mild cognitive impairment *n* = 89	Dementia *n* = 109	Test of significance
**Demographics**				
Age at baseline, mean (SD)	60.85 (8.65)	73.70 (8.02)	73.80 (9.59)	*F* = 159.55, *p* < 0.05
Women, *n* (%)	370 (66.4)	31 (34.8)	46 (42.2)	χ^2^(2) = 46.96, *p* < 0.05
Education, mean (SD)	16.00 (2.58)	15.70 (2.91)	14.24 (2.75)	*F* = 20.25, *p* < 0.05
Married, *n* (%)	392 (70.4)	70 (78.7)	87 (79.8)	χ^2^(2) = 5.88, *p = n.s.*
Able to live independently, *n* (%)	557 (100%)	63 (70.8)	20 (18.3)	χ^2^(2) = 485.98, *p* < 0.05
Lives alone, *n* (%)	80 (14.4)	9 (10.1)	11 (10.1)	χ^2^(2) = 2.30, *p = n.s.*
Race				χ^2^(4) = 9.55, *p* < 0.05
White, *n* (%)	440 (79.0)	78 (87.6)	97 (89.0)	
Black, *n* (%)	98 (17.6)	8 (9.00)	11 (10.1)	
Other, *n* (%)	19 (3.4)	3 (3.4)	1 (0.9)	
Ethnicity				χ^2^(2) = 1.28, *p = n.s.*
Hispanic, n (%)	4 (0.7%)	1 (1.1)	2 (1.8)	

### Association Between Affect and Cognitive Health Status in Full Sample

Table 2 represents the results of the analyses. In the full sample, chi-square test results revealed associations (a) between happiness and CHS, χ^2^(2) = 6.06, *p* < 0.05 (*Cramer’s V* = 0.09), (b) between helplessness and CHS, χ^2^(2) = 6.44, *p* < 0.05 (*Cramer’s V* = 0.09), and (c) between hopelessness and CHS, χ^2^(2) = 14.11, *p* < 0.01 (*Cramer’s V* = 0.14).

**TABLE 2:** Positive and negative affect in all sample, Whites, and Blacks.

Characteristics	Cognitively normal *n* = 557	Mild cognitive impairment *n* = 89	Dementia *n* = 108	Test	Effect Size
**Positive and Negative Affect in All Sample**					
Happiness (No vs. Yes), *n* (% within diagnosis)	39 (7.0) vs. 518 (93.0)	13 (14.6) vs. 76 (85.4)	10 (9.3) vs. 98 (90.7)	χ^2^(2) = 6.06, *p* < 0.05	*Cramer’s V* = 0.09*
Helplessness (No vs. Yes), *n* (% within diagnosis)	525 (94.3) vs. 32 (5.7)	79 (88.8) vs. 10 (11.2)	96 (88.9) vs. 12 (11.1)	χ^2^(2) = 6.44, *p* < 0.05	*Cramer’s V* = 0.09*
Hopelessness (No vs. Yes), *n* (% within diagnosis)	553 (99.3) vs. 4 (0.7)	86 (96.6) vs. 3 (3.4)	102 (94.4) vs. 6 (5.6)	χ^2^(2) = 14.11, *p* < 0.01	*Cramer’s V* = 0.14**

**Characteristics**	**Cognitively normal** ***n* = 440**	**Mild cognitive impairment** ***n* = 78**	**Dementia** ***n* = 96**	**Test**	**Effect Size**

**Positive and Negative Affect in Whites**					
Happiness (No vs. Yes), *n* (% within diagnosis)	31 (7.0) vs. 409 (93.0)	8 (10.3) vs. 70 (89.7)	8 (8.3) vs. 88 (91.7)	χ^2^(2) = 1.04, *p* = *n.s*	*Cramer’s V* = 0.04
Helplessness (No vs. Yes), *n* (% within diagnosis)	421 (95.7) vs. 19 (4.3)	71 (91.0) vs. 7 (9.0)	85 (88.5) vs. 11 (11.5)	χ^2^(2) = 8.47, *p* < 0.05	*Cramer’s V* = 0.12*
Hopelessness (No vs. Yes), *n* (% within diagnosis)	439 (99.8) vs. 1 (0.2)	76 (97.4) vs. 2 (2.6)	91 (94.8) vs. 5 (5.2)	*Fisher-Freeman-Halton < 0.05*	*Cramer’s V* = 0.16**

**Characteristics**	**Cognitively normal** ***n* = 98**	**Mild cognitive impairment** ***n* = 8**	**Dementia** ***n* = 11**	**Test**	**Effect size**

**Positive and Negative Affect in Blacks**					
Happiness (No vs. Yes), *n* (% within diagnosis)	8 (8.2) vs. 90 (91.8)	3 (37.5) vs. 5 (62.5)	2 (18.2) vs. 9 (81.8)	Fisher-Freeman-Halton *< 0.05*	*Cramer’s V* = 0.25***
Helplessness (No vs. Yes), *n* (% within diagnosis)	88 (89.8) vs. 10 (10.2)	7 (87.5) vs. 1 (12.5)	10 (90.9) vs. 1 (9.1)	Fisher-Freeman-Halton *= n.s.*	*Cramer’s V* = 0.02
Hopelessness (No vs. Yes), *n* (% within diagnosis)	95 (96.98) vs. 3 (3.1)	7 (87.5) vs. 1 (12.5)	10 (90.9) vs. 1 (9.1)	Fisher-Freeman-Halton *= n.s.*	*Cramer’s V* = 0.14

*Small effect size, **Small-to-moderate effect size, ***Large effect size.

### Association Between Affect and Cognitive Health Status in Black and White Participants

When the relationship between affect and CHS was examined by race, the association of happiness with CHS remained significant in the Black sample (Fisher-Freeman-Halton exact < 0.05; *Cramer’s V* = 0.25), but not in Whites, χ^2^(2) = 1.04, *p* = *n.s*. Both dimensions of negative affect also revealed differences by race. The association of helplessness with CHS was not significant for Blacks (Fisher-Freeman-Halton exact = *n.s*.), but was significant for Whites, χ^2^(2) = 8.47, *p* < 0.05 (*Cramer’s V* = 0.12). Finally, hopelessness and CHS were not significantly related in Blacks (Fisher-Freeman-Halton exact = *n.s.*), but were in Whites, Fisher-Freeman-Halton exact < 0.05 (*Cramer’s V* = 0.16).

## Discussion

This study examined whether happiness, helplessness, and hopelessness are linked cross-sectionally to cognitive status in a well-characterized sample, and whether these associations differ by race. Consistent with previous research evidence, we found that happiness, helplessness, and hopelessness were associated with cognitive health status in our total group of participants. In our large, racially diverse sample, we observed that significantly smaller percentage of older adults with dementia (90.7%) reported happiness compared to cognitively normal participants (93%). Interestingly, we found that a significantly smaller percentage of people with MCI (85.4%) reported happiness compared to cognitively normal participants and those with dementia. We also observed that greater percentages of participants with MCI and dementia endorsed helplessness and hopelessness when compared to cognitively normal participants. This finding is in agreement with earlier reports that negative affect was related to worse cognitive health (Danhauer et al., 2013; Gates et al., 2014). Although not longitudinal data, these associations suggest CHS and mood associate in an incremental manner.

Our second goal was to explore whether associations between affect and cognitive health status differed by race. Conversely, both helplessness and hopelessness (negative affect) were related to cognitive health status in Whites but not in Blacks. These findings echo the Assari and Lankarani (2016) findings that the association between negative affect and chronic health conditions are stronger for Whites compared to their Black counterparts.

Interestingly, there was a non-linear relationship between happiness and CHS in Black but not in White participants. Similar to the pattern observed in the total sample, Black participants with mild cognitive impairment were significantly less likely to report feeling happy compared to either the cognitively healthy or dementia groups. Although the number of Black study participants with MCI or dementia was considerably smaller than the comparable White subgroup, the effect size of the inverted U-shaped relationship observed in the Black participant subgroup was large. Stites et al. (2017) described an interesting finding wherein participants with MCI expressed lower wellbeing than the cognitively normal and Alzheimer’s disease dementia groups. The authors further suggested that the relationship between quality of life and cognition could be influenced by individual’s expectations of prognosis and the diagnostic label. Applied to our data, this would suggest our findings resulted from participants’ insights about their diminishing cognitive status. Specifically, the finding that individuals with MCI (85.4%) were less likely to endorse happiness compared to those who were cognitive healthy (93.0%) and individuals with dementia (90.7%). Moreover, those with MCI (11.2%) were more likely to endorse helplessness compared to those denoted as cognitively normal (5.7%) and diagnosed with dementia (11.1%).

In particular, those who were aware of their diagnosis reported lower quality of life and great cognitive difficulties than those who were not aware. These findings warrant further exploration. If replicated, this suggests that one’s awareness of diagnosis plays a role in positive and negative affect. For example, those with MCI may feel more anxiety and sadness due to their projected prognosis. Future research could examine whether participants’ degree of insight moderates the relationship between their condition and positive and negative affect.

There are a few limitations that should be considered when interpreting this study’s findings. First, this study was conducted using a cross-sectional data, limiting our ability to identify any directionality and causality of the associations among variables. Future research with longitudinal data may determine the directionality and causality of the associations among the variables. Participants were mainly White and recruited from the Wisconsin ADRC, which may limit the generalizability of this study’s findings to other areas of the US. In this study, we only compared differences between Blacks and Whites. Future studies should explore how happiness, helplessness, and hopelessness may vary across other racial or ethnicity groups. Additionally, racialized identity in the United States is associated with other social factors, such as socioeconomic disadvantage, healthcare disparities, and discrimination. The challenges deriving from these psychosocial and socioeconomic factors could adversely affect relative levels of positive and negative affect. Future studies would benefit from further consideration of these factors and how they might impact the associations between CHS, race/ethnicity, and affect. More rigorous survey measurements (e.g., multiple items for constructs) may also provide deeper insight into the association between affect and cognitive health status. For example, use of a measure such as the Positive Affect and Negative Affect Schedule (PANAS) (Watson et al., 1988) would allow for multi-dimensional examination of affect. Additionally, there may be measurement effects as people with various cultural backgrounds may have interpreted and answered the questions differently. It may be more acceptable to acknowledge negative affect in White culture. Although, Wyman et al. (2019) found that depressive symptoms were the same in Black and White older women, the women’s endorsement of impairment was very different and led to different rates of formal diagnosis.

This study is also limited in that we did not examine the intersectionality of race/ethnicity and gender. Aspects of one’s identity are marginalized in varying degrees, which in turn, effects the relationship between affect and cognitive health status. For example, McIntosh and Danigelis (1995) observed interesting patterns of influence for paid and unpaid productive activity on positive and negative affect, when mood was assessed among older Black and White male and female adults. Although paid work did not affect either positive or negative affect for any of the participants, Black women relied on formal religious participation for reducing negative affect, and informal volunteering increased positive affect and reduced negative affect for both Black men and White women. Formal non-religious volunteering was most important for increasing positive affect among older White men and reducing negative affect of older Black men. Another limitation is that dementia type was not taken into consideration in our analysis. This is an important limitation given qualitative and quantitative differences in mood/affect issues can be detected across groups of patients affected by different neurodegenerative conditions. Future research can consider dementia types in their data analysis. Finally, we did not use demographic variables such as age and gender as covariate variables. For example, participants’ mood may change based on their age and age-related factors which we did not take into consideration in this study. Therefore, future studies with larger samples may take into consideration demographic variables.

## Conclusion

In conclusion, this study provided support for the association of both positive and negative affect with cognitive health status in middle- to older-aged adults. Further, this association varied by race, such that a dimension of positive affect was associated with cognitive health status in Blacks but not in Whites, and dimensions of negative affect were associated with cognitive health status in Whites but not in Blacks. These associations warrant replication. Nonetheless, the present study is an advance for the areas of health and aging because it provides ample evidence of the ways in which cognitive and affective health are related. The present exploratory study succeeded in initiating the much-needed dialogue on racial/ethnic differences in the association between affect and CHS. We look forward to having sufficiently large samples of different racial/ethnic groups as well as gender representation to explore these associations further.

## Data Availability Statement

The datasets presented in this article are not readily available because data request should be directly made to the Wisconsin Alzheimer’s Disease Research Center at the University of Wisconsin-Madison. Requests to access the datasets should be directed to the Wisconsin Alzheimer’s Disease Research Center at adrc@medicine.wisc.edu.

## Ethics Statement

The studies involving human participants were reviewed and approved by the University of Wisconsin-Madison. The patients/participants provided their written informed consent to participate in this study.

## Author Contributions

All authors listed have made a substantial, direct, and intellectual contribution to the work, and approved it for publication.

## Conflict of Interest

The authors declare that the research was conducted in the absence of any commercial or financial relationships that could be construed as a potential conflict of interest.

## Publisher’s Note

All claims expressed in this article are solely those of the authors and do not necessarily represent those of their affiliated organizations, or those of the publisher, the editors and the reviewers. Any product that may be evaluated in this article, or claim that may be made by its manufacturer, is not guaranteed or endorsed by the publisher.
